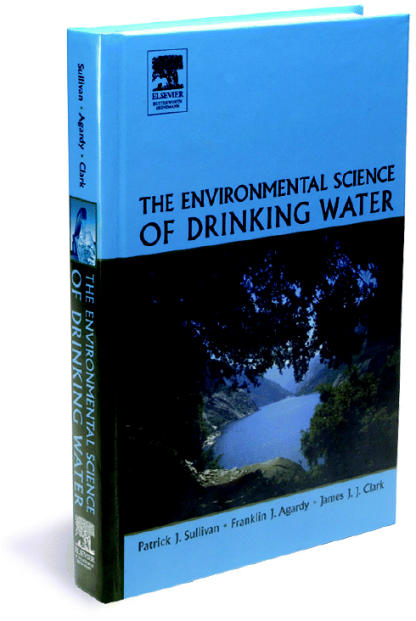# The Environmental Science of Drinking Water

**Published:** 2005-12

**Authors:** Shawna Bourne

**Affiliations:** Shawna Bourne is a Certified Inspector of Public Health and works for the Ontario Ministry of the Environment where she manages the Drinking Water Program for the London District. She is also a board member of the Stanier Institute (Linking Health to Hygiene) and is an executive on behalf of the Ontario Branch Members of the Canadian Institute of Public Health Inspectors.

Patrick J. Sullivan, Franklin J. Agardy, and James. J.J. Clark

Burlington, MA:Elsevier, 2005. 368 pp. ISBN: 0-7506-7876-3, $59.95 cloth

Our ever-increasing knowledge of the subtle impacts of contaminants on the ecosystem and the public health of the community demand an evidence-informed and fact-based approach to dealing with the contamination of what has been coined the “lifeblood of the planet.” The authors of *The Environmental Science of Drinking Water* question the current culture of drinking water management in the United States and ask whether the reasoning behind the current management system is a sound one—much like the Native American proverb “The frog does not drink up the pond in which he lives,” a foolish practice that would lead to its own demise; the authors conclude that we should approach the management of our water supplies as the frog does and not foul the foundation of our, thus far, successful existence.

The authors raise a red flag early, citing the innumerable, unquantified industrial chemicals, pesticides, pharmaceuticals, and treatment byproducts released into our communal waterways. Five chapters cover basic water chemistry, modern chemical contaminants found in water systems, and the treatment technologies available for contaminant control and remediation. This information is supplemented by numerous relevant appendices and a glossary of terms; unfortunately, the work is scattered with abbreviations that perplex the unfamiliar reader and make the text difficult to follow at times. The authors stress the importance of an ecosystem approach to risk management, noting that a paradigm shift is necessary to protect against the long-term synergistic effects of the “chemical soup” that our water resources are becoming.

*The Environmental Science of Drinking Water* is an excellent compilation of fact, case study, policy analysis, opinion, and good old-fashioned common sense. This enlightening book is an excellent text suitable for technical experts in the field, students honing their skills in environmental health or engineering, or laypersons interested in understanding the origins of policies developed by the U.S. government.

Unfortunately, this text quickly dismisses the significance of microbial contaminants, which is a faulty approach from my perspective. Just as chemical risks due to the contamination of water supplies have changed over time, so too have the biological risks of the water supply and the susceptibilities of the population at large who consume it. The focus on water chemistry and related risks may warrant a review of the title of this textbook, with a more appropriate title being *Drinking Water: The Chemical Risks in Modern Water Supplies*. Such a title would more accurately reflect the scope and focus of this body of excellent work.

Clearly, these problems are not restricted to the United States. Given the many great examples and case studies used throughout the text, additional benefit would be gained by considering the state of affairs around the world and both the failures and successes in the implementation of water policy; this would add to the weight of the authors’ policy arguments. Some government agencies have begun to look at the broader impact of chemical and other contamination of water supplies; for example, in Ontario, Canada, goals include leading industry beyond minimum standards and toward “environmental improvements” through incentives.

Overall, the thesis is presented methodically and comprehensively. The arguments sway the reader to conclude that waste management and water treatment technology can be used better to reduce the risk to our water supplies. Source protection and drinking water treatment are available to exact a change. The new question is how to share this knowledge with the public, articulate common values and expectations, and mobilize the populace to put its money where its mouth is. The most remarkable achievement of this book is that it has the ability to move these and other important questions to the forefront of the minds of those who read it and begin that paradigm shift through the discourse of reason.

## Figures and Tables

**Figure:**